# Measuring women’s experiences of decision-making and aspects of midwifery support: a confirmatory factor analysis of the revised Childbirth Experience Questionnaire

**DOI:** 10.1186/s12884-020-02869-0

**Published:** 2020-04-06

**Authors:** Anna Dencker, Liselotte Bergqvist, Marie Berg, Josephine T. V. Greenbrook, Christina Nilsson, Ingela Lundgren

**Affiliations:** 1 Institute of Health and Care Sciences, Sahlgrenska Academy, University of Gothenburg, Box 457, SE-405 30 Gothenburg, Sweden; 2 Department of Obstetrics, Sahlgrenska University hospital, Gothenburg, Sweden; 3 Mason Institute of Medicine, Life Science and the Law, University of Edinburgh, Edinburgh, UK; 4 Faculty of Caring Science, Work Life and Social Welfare, University of Borås, Borås, Sweden

**Keywords:** Childbirth experience questionnaire, CEQ, CEQ2, Maternal satisfaction

## Abstract

**Background:**

Women’s experiences of labour and birth can have both short- and long-term effects on their physical and psychological health. The original Swedish version of the Childbirth Experience Questionnaire (CEQ) has shown to have good psychometric quality and ability to differentiate between groups known to differ in childbirth experience*.* Two subscales were revised in order to include new items with more relevant content about decision-making and aspects of midwifery support. The aim of the study was to develop new items in two subscales and to test construct validity and reliability of the revised version of CEQ, called CEQ2.

**Method:**

A total of 11 new items (*Professional Support* and *Participation*) and 14 original items from the first CEQ (*Own capacity* and *Perceived safety*), were answered by 682 women with spontaneous onset of labour. Confirmatory factor analysis was used to analyse model fit.

**Results:**

The hypothesised four-factor model showed good fit (CMIN = 2.79; RMR = 0.33; GFI = 0.94; CFI = 0.94; TLI = 0.93; RMSEA = 0.054 and PCLOSE = 0.12) Cronbach’s alpha was good for all subscales (0.82, 0.83, 0.76 and 0.73) and for the total scale (0.91).

**Conclusions:**

CEQ2, like the first CEQ, yields four important aspects of experience during labour and birth showing good psychometric performance, including decision-making and aspects of midwifery support, in both primiparous and multiparous women.

## Background

Women’s experiences of labour and birth can have both immediate and long-term effects on their overall health. Positive experiences are important in both normal and complicated childbirth, to empower women physically, psychologically, and socially [1, 2]. A positive birthing experience can give women feelings of being innately empowered and strengthened, leaving them encouraged in the face of impending motherhood. Positive birth experiences have been found to relate to both internal and external factors, such as underlying sources of personal strength, and sense of control and coherence, as well as interpersonal interactions with health care professionals displaying welcoming and supportive behaviours in perinatal encounters [1–4].

Conversely, negative experiences have been associated with deterioration of maternal health, inducing somatic symptoms and psychological morbidity [5, 6]. A negative birth experience [6] has been identified as an important factor in delaying or avoiding subsequent pregnancies, and in requesting an elective caesarean [7–9]. Furthermore, reports of traumatic birth experiences have been related to the development of post-partum depression (PPD), post-traumatic stress disorder (PTSD), anxiety, and fear of childbirth (FOC) [10–13], as well as problematic bonding between mother and infant, leading to the development of disorganised attachment in the child [14].

Measuring experiences relating to labour and birth is beneficial. Quantifying important elements of women’s experiences can assist clinicians in evaluating their own practice. Furthermore, documenting dissatisfaction is important in ensuring women are provided high quality care during labour and birth. Healthcare professionals’ engagement in the reciprocal relationship with women and their partners, the promotion of feelings of safety, security, and comfort in the birthing environment, and the enhancement of trust and confidence in professional competence and knowledge are all important elements needed in order for women to experience childbirth positively [15, 16].

Whilst many tools measuring women’s perinatal experiences exist, most scales lack appropriate psychometric validation. Furthermore, most scales focus on specific areas of the pre-labour or birthing experience [17]. Due to the lack of a more all-encompassing instrument, the multidimensional Childbirth Experience Questionnaire (CEQ) was developed in Sweden, with items derived from patient interviews and discussions with medical professionals, which were then categorised into four domains [18]. The instrument was found to be robust and reliable, discriminating well between groups previously found to differ substantially in birthing experience (e.g. those with instrumental births, those in labour in excess of 12 h, and those who had their labours augmented with oxytocin). The CEQ has since been translated and validated in several languages [19–23], and has been successfully implemented in empirical studies including a large variety of culturally diverse samples [24–30]. However, measurement instruments always hold potential for improvement, and in validating the original Swedish version of the instrument, two domains showed weaker performance; *Participation* and *Professional Support* [18]. Thus, the aim of the present study was to revise the CEQ by developing new items in two subscales, and to test construct validity and reliability of the novel version, CEQ2.

## Methods

### Childbirth experience questionnaire, CEQ

The original CEQ, validated in Sweden in 2010 [18], yields 22 items in four domains: *Own capacity*, *perceived safety*, *professional support*, and *participation*. Responses are scored using a 4-point Likert scale ranging from 4 (totally agree), 3 (mostly agree), 2 (mostly disagree), to 1 (totally disagree). Three items referring to labour pain, sense of security and control are assessed with visual analogue scales (VAS). The VAS-scales scores are transformed to categorical values, 0–40 = 1, 41–60 = 2, 61–80 = 3 and 81–100 = 4. A higher score equals a better experience. Negatively worded items are reversed in scoring, including the pain item [18]. Test-retest reliability and criterion validity of the CEQ are reassuring [19] and the model has been tested with a confirmatory factor analysis [20].

New items covering the domains of *participation* and *professional support* were developed for the present study, to be tested together with the original items included in *own capacity* (8 items) and *perceived safety* (6 items).

### Development of new items

Interviews with childbearing women, discussions with two specialist midwives (of which one worked with women with a fear of childbirth), and reading of emergent empirical literature in the study field, were used in adding content and revising the items in the domains of *professional support* and *participation* in the original CEQ [18]. In *professional support,* reversed items were developed in order to avoid high ceiling effects. In *participation,* more relevant items relating to information and decision-making were added. In total, following discussion with experts in midwifery science, and after being scrutinised for language clarity, 14 new items were developed for psychometric testing.

### Face validity

A group of eight women in the postpartum period and one experienced midwife were used to test the face validity of the total item set; 14 items from the original CEQ, and 14 newly developed items. One co-author (LB), who is a midwife, interviewed Swedish speaking women (including one who was not a native Swedish speaker) when they presented for postnatal follow-up visit (*n* = 6), as well as women with fear of childbirth (*n* = 2) when they came for follow-ups at the clinic with a midwife specialist in counselling. The respondents had various birth outcomes: Five women had a normal birth, two women had an emergency caesarean during the second stage of labour when they were fully dilated, and one woman had a retained placenta with extensive haemorrhaging. The women answered the items, and were asked further questions about every item. During the interviews, each woman was asked to rate if the items were relevant for their birth experience, and whether they felt the items were easy to understand. All the women were also asked to identify items of special importance to them.

In testing face validity, three of the newly developed items were identified as ambiguous, and were therefore subsequently rejected; leaving 11 new items for further testing. Through face validity testing, these remaining items were deemed easy to understand and relevant to the group of postpartum women. However, to enhance clarity, minor linguistic adjustments to the Swedish language were made prior to further testing.

### Participants and data collection

Data collection was conducted at three maternity departments at Sahlgrenska University Hospital in Gothenburg, Sweden, between January and May of 2015. When the women presented for follow-up, 3–5 days after childbirth, they were informed of the study and asked if they wished to participate. Those who were interested in participating provided their email address. An invitation letter with information was sent three to four weeks postpartum to all women who agreed to participate, including a link to an online questionnaire. This questionnaire consisted of 25 items; 14 original items and 11 newly developed items, for psychometric testing. Further, the questionnaire included questions regarding the women’s age, number of children, onset of and duration of labour, mode of birth, and previous caesarean. A total of 682 women with spontaneous onset of labour (women with induction and elective caesarean excluded) answered the email invitation.

### Psychometric testing

Confirmatory factor analysis was applied to test the factor structure using AMOS software (SPSS version 25). Cronbach’s alpha was computed for subscales and total scale. Scores were subsequently compared between groups known to differ in childbirth experience (parity, mode of birth, oxytocin treatment, and length of labour).

## Results

A total of 682 women with spontaneous onset of labour (62.7% response rate) completed the CEQ2 questionnaire, three to 4 weeks postpartum. For the confirmatory factor analysis, responses from a subset of women with no missing answers (*n* = 615) were used. Near half of the women were primiparous (46.9%) and 52.6% were multiparous. Mean age was 31.4 years. Respondent characteristics in both the total and the subset sample are shown in Table 1.

**Table 1:** Characteristics of the study population

Variables	Total sample ***n*** = 682	Subsample in CFA ***n*** = 615
Maternal age, years, mean (SD)	31.4 (4.6)	31.4 (4.6)
Number of births, mean (SD)	1.69 (0.8)	1.67 (0.8)
Primiparous	320 (46.9)	293 (47.6)
Multiparous	359 (52.6)	319 (51.9)
Oxytocin augmentation	179 (26.2)	161 (26.2)
Active labor, duration > 12 h	175 (25.7)	156 (25.4)
Spontaneous vaginal birth	596 (87.4)	542 (88.1)
Instrumental vaginal birth	39 (5.7)	36 (5.9)
Emergency caesarean	46 (6.7)	37 (6.0)

Data are given as as n (%) or mean (SD)

*CFA* Confirmatory Factor Analysis

### Confirmatory factor analysis

The hypothesised four-factor model was tested through a confirmatory factor analysis on a subset of women with spontaneous onset of labour and with no missing answers (*n* = 615). A 22-item version showed good model fit in the four-factor model; CMIN = 2.79; RMR = 0.33; GFI = 0.94; CFI = 0.94; TLI = 0.93; RMSEA = 0.054 and PCLOSE = 0.12. The CEQ2 model with four subscales; *own capacity* (8 original items), *perceived safety* (6 original items), *professional support* (5 new items), and *participation* (3 new items) was confirmed. Three new items did not fit in the model and were therefore excluded, see Table 2.

**Table 2:** Confirmed model of 4 subscales/domains and items in CEQ2

Item	New item	Reversed item
**Own capacity (8 items)**		
Labour and birth went as I had expected.		
I felt strong during labour and birth.		
I felt capable during labour and birth.		
I felt happy during labour and birth.		
I felt that I handled the situation well.		
I was tired during labour and birth.		R
As a whole, how painful did you feel childbirth was? ^a^		R
As a whole, how much control did you feel you had during childbirth? ^a^		
**Perceived safety (6 items)**		
I felt scared during labour and birth.		R
My impression of the team’s medical skills made me feel secure.		
I have many positive memories from childbirth.		
I have many negative memories from childbirth.		R
Some of my memories from childbirth make me feel depressed.		R
As a whole, how secure did you feel during childbirth? ^a^		
**Professional support (5 items)**		
Both my partner and I were treated with warmth and respect.	X	
I would have preferred the midwife to be more present during labour and birth.	X	R
I would have preferred more encouragement from the midwife.	X	R
The midwife conveyed an atmosphere of calm	X	
The midwife helped me to find my inner strength	X	
**Participation (3 items)**		
I wish the staff had listened to me more during labour and birth.	X	R
I took part in decisions regarding my care and treatment as much as I wanted.	X	
I received the information I needed during labour and birth.	X	

^a^Visual analogue scale (VAS)

Three items were excluded after the analysis; I would have preferred another form of pain relief, I could get up and move around as much as I wanted, I could give birth in the way I wanted

### Internal consistency

Reliability with Cronbach’s alpha was satisfying for all subscales (*own capacity* 0.82, *perceived safety* 0.83, *professional support* 0.76, *participation* 0.73) and for the total scale (0.91), see Table 3.

**Table 3:** Descriptive statistics for subscale and total scale scores, n = 682

Domain	Mean (SD)	Min	Max	Cronbach’s alpha
Own capacity	2.80 (0.56)	1.13	3.88	0.82
Perceived safety	3.35 (0.62)	1.00	4.00	0.83
Professional support	3.59 (0.52)	1.00	4.00	0.76
Participation	3.45 (0.58)	1.00	4.00	0.73
Total scale	3.30 (0.47)	1.38	4.00	0.91

### Known-groups validity

Discriminant validity of the CEQ2 was tested using Mann-Whitney U-test, to compare score levels between groups known to differ in birthing experience. Scores were higher in multiparous women, in women with spontaneous vaginal birth, in women without oxytocin treatment for slow labour progress, and in women with labour duration less than 12 h. No significant differences were seen between age groups (Table 4).

**Table 4:** Differences in subscale and total scores between groups, mean (SD), n = 682

Group	n	Own capacity	Perceived safety	Professional support	Participation	Total score
Parity						
Primiparous	308	2.67 (0.54)	3.27 (0.68)	3.56 (0.58)	3.37 (0.64)	3.22 (0.50)
Multiparous	341	2.92 (0.53)	3.43 (0.57)	3.61 (0.45)	3.53 (0.51)	3.38 (0.42)
*P*-value ^a^		< 0.001	0.003	0.960	0.001	< 0.001
Maternal age						
≤ 30 years	271	2.77 (0.55)	3.33 (0.62)	3.57 (0.57)	3.54 (0.60)	3.28 (0.47)
> 30 years	381	2.82 (0.56)	3.37 (0.62)	3.60 (0.49)	3.46 (0.56)	3.32 (0.46)
P-value ^a^		0.181	0.258	0.559	0.738	0.212
Labor duration						
≤ 12 h	481	2.90 (0.54)	3.45 (0.57)	3.63 (0.47)	3.50 (0.54)	3.37 (0.43)
> 12 h	168	2.51 (0.51)	3.07 (0.69)	3.46 (0.61)	3.32 (0.64)	3.09 (0.50)
P-value ^a^		< 0.001	< 0.001	0.003	< 0.001	< 0.001
Oxytocin treatment						
Yes	172	2.50 (0.53)	3.13 (0.64)	3.51 (0.56)	3.36 (0.59)	3.12 (0.46)
No	463	2.92 (0.52)	3.45 (0.58)	3.63 (0.49)	3.49 (0.56)	3.37 (0.44)
P-Value ^a^		< 0.001	< 0.001	0.012	0.002	< 0.001
Mode of birth						
Spontaneous vaginal	568	2.86 (0.54)	3.41 (0.59)	3.61 (0.51)	3.50 (0.57)	3.34 (0.45)
Instrumental ^b^	82	2.41 (0.54)	2.98 (0.70)	3.44 (0.57)	3.16 (0.60)	3.00 (0.48)
P-value ^a^		< 0.001	< 0.001	< 0.001	< 0.001	< 0.001

^a^Mann-Whitney U-test

^b^Instrumental vaginal birth + emergency caesarean

## Discussion

Health-focused woman-reported outcomes, such as childbirth experience [31], are important to use alongside clinical/medical outcomes, in order to measure quality of treatment and care from the patient’s perspective [32]. In adding more aspects of women’s childbirth experience, the present study resulted in the development of the CEQ2, a revised version of the CEQ. Like the original CEQ [18] the CEQ2 has 22 items in the same four hypothesised domains, showing satisfying Cronbach’s alphas and ability to discriminate between known groups. These results indicate that the CEQ2, like the original CEQ [19, 20], shows good psychometric properties and can be applied in assessing women’s birthing experiences.

Both the CEQ and the CEQ2 were developed in Sweden, and have been subsequently translated into several languages. In parallel with the present study, two other validation studies of translated versions of the CEQ2 have been published. In the United Kingdom, the English version of the CEQ2 showed good psychometric properties when testing face validity, criterion validity in relation to the nationally used Maternity Survey, test-retest reliability and differences between known groups. Cronbach’s alphas were satisfying, except for the *participation* subscale [33]. In Iran, a Farsi version of the CEQ2, with 23 items (including the mobility item, about being able to get up and move around during childbirth), showed good model fit in a confirmatory factor analysis [34].

Regarding content validity of the instrument; three of four domains in the CEQ2, as in the original CEQ, are consistent with previous research reporting on important aspects of women’s satisfaction with care received during childbirth. Several important factors influence the overall birth experience [35], including: compassion and understanding (addressed in the domain *professional support*); competency (addressed in the domain *perceived safety*); privacy, dignity, and respect (addressed in the domain *professional support*); receiving information and involvement in decision-making (addressed in the domain *participation*). A positive experience of *perceived safety* is central, specifically for primiparous women, in order to avoid the development of negative memories and fear of a future pregnancy and birth [5, 6, 10]. The domain of *own capacity* reflects women’s assessment of their own agency, strengths, emotions, and bodily sensations during birth, rather than their experiences of the treatment received in their encounters with health care professionals [1, 32, 36]. Furthermore, and demonstrating the complexity of the birth experience [32], all four domains of the revised CEQ2 are inherently interrelated, as how women are treated and cared for by health care professionals during childbirth impacts their assessments of childbirth experience [37].

Both the original CEQ and the CEQ2 questionnaires have a solid ground for content validity, as they are founded on focus group interviews with the target group of childbearing women, as well as with experts in midwifery and obstetrics (midwives, an obstetrician for the first CEQ), and a midwife counsellor (working with women with a fear of childbirth) [18]. Cronbach’s alphas and differences between known groups show good measurement reliability, and ability to detect change [38]. Prior to revising the instrument, it was decided to keep the two domains from the original CEQ showing the best psychometric performance; *own capacity* and *perceived safety*, and to develop new items in the effort to enhance the domains of *participation* and *professional support.* Whilst these domains were considered important to revise due to their weaker psychometric ability [18], the relevance of such efforts is further accented by indications that both overall birth experience and medical outcomes are affected by women’s experiences of participation and the quality of the support received from health care professionals in conjunction with childbirth [9, 39, 40]. Furthermore, professional support and participation are central dimensions in midwifery care, encapsulating the midwife-woman relationship and woman-centred care [41], as well as in midwifery models of care [15, 42].

The new items in the *participation* domain were intentionally developed to address aspects relating to receiving information, involvement in decision-making, and perceptions of being heard during labour and birth [1]. This reflects an ongoing shift away from clinician-led care, toward woman-centred approaches to care provision during childbirth [15, 42]. Corresponding items in the *participation* domain in the original CEQ assessed possibilities to be up and free to move, and to select birthing position and pain relief. Women’s choices of mobility and upright positions [43] during labour are beneficial, and should be encouraged [44]. Whilst one newly developed item addressing mobility did not fit in the yielded model, and was thus excluded in the CEQ2, new items relating to receiving information and involvement in decision-making were identified to be of greatest importance and relevance to include [45].

Including altered wording, the new items in the *professional support* subscale in the CEQ2 redirect focus, away from evaluating the midwife as in the original CEQ [18], to instead assess the woman’s experience of her personal needs for support during labour and birth. Respectful care during childbirth is paramount [46], and disrespectful care is related to a very negative birthing experience [47]. Thus, a newly developed item addressing experiences of being met with respect was also included.

The second excluded item was worded “*I could give birth in the way I wanted*”. This specific item correlated with all domains in the CEQ2, and thus seemed to reflect an overall experience, more so than a specific domain of the experience. The third excluded item addressed being able to be up and mobile, an important aspect during labour and birth [44]. Whilst the original CEQ included a corresponding item [18], the CEQ2 focuses more explicitly on other elements of women’s right to participation during labour and birth, accenting their right to information, their right to involvement in decision-making, and their right to be heard.

### Strengths and limitations

Notwithstanding the aforementioned focused efforts to further develop and refine an all-encompassing instrument, multidimensional scales have limitations, and included items must adhere to the rules of measurement theory [38]. Guided by this principle, three items were excluded in the analysis, due to misfit to the model. One of the excluded items related to preferred pain relief. Whilst important, an assessment of overall pain during labour and birth is already included in the domain of *own capacity*, and therefore included in the scale. Experienced labour pain is complex, and difficult to explicitly relate to childbirth experience, as many women who experience high levels of pain may still report a positive birthing experience [48]. Conversely, reporting a very high level of pain a month or more following childbirth is often related to a negative experience [48], and constitutes a risk factor for having experienced birth trauma, as well as an increased risk for developing fear of childbirth [10].

Limitations of the present study include persistent ceiling effects in the *professional support* domain, especially in the item addressing being met with respect. Whilst being treated with respect is an important aspect of childbirth [49], these ceiling effects indicate the need for further refining items. Additionally, merging respect conveyed toward both the woman and their partner in one item may be a methodological flaw, and may render the item difficult to answer for some women. Whilst the observed ceiling effects counter this argument, the sensitivity of the item could be enhanced by distinguishing targets more explicitly. Notwithstanding these limitations, the strengths of this study include a thorough groundwork in item development, building on an existing and well validated instrument [18–21], and the inclusion of items addressing decision-making and aspects of midwifery support. A further strength is that the CEQ2 is also validated for multiparous women.

## Conclusions

Several measures can be used to assess experiences during pregnancy and childbirth, however, few consist of multidimensional and validated scales. The CEQ2, as the original CEQ, yields four important aspects of experience during labour and birth (*own capacity*, *perceived safety*, *professional support*, and *participation*), shows good psychometric performance, and is therefore useful in measuring women’s assessments of childbirth experience, including decision-making and aspects of midwifery support, in both primiparous and multiparous women.

## Data Availability

The dataset used in the present study is available from the corresponding author upon reasonable request.

## Abbreviations

CEQ: Childbirth Experience Questionnaire
CEQ2: Childbirth Experience Questionnaire revised version
CFI: Comparative Fit Index
CMIN: Chi-square goodness of fit
FOC: Fear of Childbirth
GFI: Goodness of Fit Index
PCLOSE: Test of fit, NS means good fit
RMR: Root Mean square Residual
RMSEA: Root Mean Square Error of Approximation
TLI: Tucker-Lewis Index
